# Deep learning framework for predicting measurement error drift in smart meter sensors under harsh coastal environments

**DOI:** 10.1371/journal.pone.0355304

**Published:** 2026-08-05

**Authors:** Tianfu Huang

**Affiliations:** 1 State Grid Fujian Marketing Service Center, Fuzhou, China; 2 Putian Coastal Atmospheric Environment Material Corrosion and Electric Power Equipment Safety Observation and Research Station of Fujian Province, Putian, China; University of Oxford, UNITED KINGDOM OF GREAT BRITAIN AND NORTHERN IRELAND

## Abstract

Smart meter sensors in coastal environments suffer severe accuracy degradation due to salt fog corrosion, high humidity, and temperature cycling affecting current transformers, voltage dividers, and measurement circuits. This study proposes a reliability prediction framework addressing multi-stress degradation in harsh marine atmospheres. The framework integrates an enhanced Transformer with multi-scale attention, a Bidirectional Long Short-Term Memory (BiLSTM) network for local degradation patterns, and a standard Transformer, unified through particle swarm optimization for dynamic weight adjustment. Validation using six years of field data comprising 10.5 million records from 200 sensors on Meizhou Island, China, demonstrates superior performance, achieving a coefficient of determination of 0.944 and a root mean square error of 0.0121%, representing a 6.9% improvement over the best individual model. To capture coastal-specific effects, two novel indices are introduced: the Salt Fog Corrosion Index, quantifying cumulative chloride deposition, and the Electrochemical Activity Factor, modeling electrochemical corrosion potential. Feature analysis identifies load current-temperature interaction with correlation 0.76 as the dominant drift mechanism, consistent with Joule heating theory. Field deployment verifies the framework’s ability to predict failures about 5 days in advance with 0.01 percentage point accuracy, enabling condition-based maintenance and reducing out-of-specification operation time by 85%. This research provides practical tools for sensor reliability in harsh environments beyond smart metering.

## Introduction

Smart meter sensors, integrating current transformers, voltage dividers, analog-to-digital converters, and electronic measurement circuits, serve as the foundational sensing infrastructure for modern intelligent distribution systems [1,2]. These multi-component sensing systems are highly vulnerable to environmental stresses, where measurement accuracy degradation directly influences electricity trading fairness, revenue protection, and power grid safety [3,4]. In coastal environments, the synergistic effects of salt fog deposition, high humidity, and thermal cycling create unique degradation pathways that accelerate sensor failure through multiple physical mechanisms including electrochemical corrosion of metallic components, insulation resistance decay, and magnetic core property alteration [5,6]. By the end of 2023, China had deployed over 650 million smart meter sensors, with more than 80 million units installed in coastal regions [7]. The accuracy degradation of these sensors affects not only fair electricity market transactions but may also trigger power grid safety incidents, establishes harsh-environment sensor reliability as a critical technical challenge.

The degradation mechanisms of smart meter sensors in coastal environments differ fundamentally from standard indoor operational conditions studied in previous research [8,9]. Salt deposition on current transformer ferromagnetic cores alters magnetic permeability through chloride ion penetration, leading to nonlinear measurement characteristics that vary with load current and temperature [6]. Moisture ingress into voltage sensing circuits causes insulation resistance decay following Arrhenius-type kinetics, where the degradation rate doubles for every 10°C temperature increase above the critical humidity threshold of 75%. Electrochemical corrosion of printed circuit board (PCB) solder joints and copper traces leads to contact resistance drift, with corrosion current density proportional to chloride concentration and relative humidity [10].

Field measurements at our Meizhou Island laboratory reveal salt deposition rates reaching 90 mg/m^3^ during typhoon seasons, 30 times higher than inland industrial environments. The wet-dry cycling characteristic of marine atmospheres creates mechanical stress through repeated salt crystallization, with each cycle inducing micro-crack propagation in component packaging. These cascading multi-physics failure modes—unique to coastal exposure—necessitate specialized prediction approaches that simultaneously account for electrochemical, thermal, and mechanical degradation pathways rather than treating temperature and humidity as independent factors.

Current research on smart meter error prediction explores multiple approaches. Traditional methods include physics-based degradation models [11,12] and statistical time series analysis [13,14], but these approaches struggle with complex component interactions and fail to capture nonlinear environmental effects. Machine learning approaches demonstrate improved adaptability, with Support Vector Machines (SVM) [15] and Random Forest (RF) [16] showing effectiveness in small-sample scenarios. Recent advances in deep learning present new opportunities: Long Short-Term Memory (LSTM) networks effectively address gradient vanishing problems in sequence modeling [17], Transformer architectures achieve long-range dependency modeling through self-attention mechanisms [18], and improved architectures such as Informer [19] and Autoformer [20] enhance time series prediction performance.

In the broader sensor reliability domain, research has addressed environmental degradation across various applications. Xu et al. reviewed Internet of Things applications in marine environment monitoring, highlighting challenges in sensor data integration and wireless communication reliability [21]. Kim et al. developed explainable machine learning frameworks for maritime sensor anomaly detection, demonstrating the importance of interpretability in industrial deployments [22]. Karganroudi et al. surveyed intelligent sensors in smart factories, emphasizing predictive maintenance as a key application for sensor networks [23]. Wang and Zhao proposed multi-scale LSTM approaches for remaining useful life prediction, addressing challenges in equipment degradation modeling [24]. However, these studies typically focus on single sensor modalities under controlled conditions or specific industrial applications rather than multi-stress coastal environments.

Existing research exhibits three critical limitations when addressing smart meter error prediction in coastal environments: First, insufficient environmental adaptability exists, as most studies rely on temperate environment data and lack systematic modeling of combined high-temperature, high-humidity, and salt fog stresses, failing to reflect the specificity of coastal environments. Second, limited model generalization occurs, where single-model approaches perform well under specific conditions but lack robustness in complex coastal environments. Third, incomplete feature engineering persists, as existing methods focus primarily on individual environmental factors while overlooking synergistic effects of temperature-humidity interactions and salt fog corrosion on measurement error evolution.

The coastal environment presents unique challenges distinct from inland regions. Salt fog deposition creates conductive pathways on insulator surfaces, accelerating electrochemical corrosion of metal components. The field measurements indicate salt deposition rates reaching 90 mg/m^3^ during typhoon seasons, 30 times higher than inland areas. The synergistic effect of salt, moisture, and temperature creates accelerated degradation mechanisms: salt crystals absorb moisture, forming electrolyte solutions; high temperatures increase ion mobility, accelerating corrosion rates; and cyclic wet-dry conditions cause mechanical stress through repeated salt crystallization. These mechanisms fundamentally differ from simple temperature or humidity effects studied in previous research, necessitating specialized modeling approaches for sensor reliability engineering.

To address these challenges, this paper proposes a deep learning ensemble framework specifically designed for smart meter sensor measurement error prediction in coastal environments. The main contributions are as follows:

1. **Multi-stress sensor degradation modeling**: Introduces Salt Fog Corrosion Index (SFCI) and Electrochemical Activity Factor (EAF) to quantify the cumulative effects of chloride deposition, moisture absorption, and temperature on sensor measurement drift. These physics-informed indices, validated through six years of field exposure data with 90 mg/m^3^ salt fog, 85% RH, and 38°C, provide the first quantitative framework for predicting sensor reliability in coastal conditions.
2. **Sensor-aware deep learning architecture**: Develops an enhanced Transformer with hybrid position encoding and multi-scale attention mechanisms specifically designed for non-stationary sensor drift patterns. The architecture achieves 66% computational reduction compared to standard Transformers while improving prediction accuracy by 6.9%, addressing the challenge of modeling both gradual drift and abrupt failure modes in sensing systems.
3. **Predictive maintenance framework for sensor networks**: Demonstrates 5–7 day advance prediction of sensor calibration requirements with 0.01% accuracy, enabling transition from time-based with 12-month intervals to condition-based maintenance strategies. Field validation shows 85% reduction in out-of-specification sensor operation time and 30% reduction in maintenance costs.
4. **Large-scale field validation and deployment guidelines**: Validates the framework using 10.5 million measurements from 200 sensors across extreme coastal conditions, identifying load current-temperature interaction with correlation strength 0.76 as the dominant drift mechanism. Feature importance analysis provides actionable design recommendations including thermal management requirements, conformal coating specifications, and optimal sensor placement strategies for harsh environment deployments.

The remainder of this paper is organized as follows: Section 1 reviews related work on sensor degradation and smart meter measurement error prediction; Section 2 details the proposed deep learning ensemble framework, including model architecture, feature engineering, and optimization strategies; Section 3 presents experimental results with comprehensive analysis; Section 4 concludes the paper and outlines future directions.

## 1. Related work

Research on smart meter measurement error prediction intersects multiple disciplines including sensor reliability engineering, environmental degradation science, and time series forecasting. This section reviews relevant progress from four perspectives: sensor degradation in harsh environments, environmental impact studies on measurement accuracy, traditional prediction methods, and deep learning approaches.

Environmental stress effects on sensor performance have been extensively studied across various industrial applications, with particular attention to marine and coastal environments where combined stresses accelerate component degradation.

Xu et al. conducted a comprehensive review of Internet of Things applications in marine environment monitoring, identifying key challenges in sensor data integration, wireless communication reliability, and long-term stability under saltwater exposure [21]. Their analysis highlighted that marine sensors face unique degradation mechanisms including bio-fouling, salt crystallization, and pressure cycling that differ fundamentally from terrestrial applications. The study emphasized the need for specialized calibration strategies and predictive maintenance frameworks for marine sensor networks.

Electrochemical corrosion in salt-laden atmospheres has been characterized by Yi et al., who investigated AISI 316L stainless steel behavior under salt spray conditions [6]. Their experimental work demonstrated that salt fog concentration and relative humidity create synergistic corrosion effects, with corrosion rates increasing exponentially when humidity exceeds 75%. The research identified that conductive salt layers facilitate galvanic corrosion between dissimilar metals, a mechanism directly relevant to smart meter PCB assemblies containing copper traces, aluminum capacitors, and solder joints.

Thermal cycling effects on electronic interconnections were examined by Noh and Jung, who studied Sn-Cu solder joint reliability under temperature cycling [10]. Their findings revealed that interfacial intermetallic compound growth accelerates under thermal stress, leading to mechanical weakening and eventual joint failure. For smart meter sensors in coastal regions experiencing diurnal temperature variations of 20–30°C, these thermal fatigue mechanisms become significant contributors to measurement drift.

Kim et al. developed an explainable machine learning framework for anomaly detection in maritime sensor data, emphasizing the importance of interpretability in industrial applications [22]. Using SHapley Additive exPlanations (SHAP) values, their approach identified which sensor variables contributed most to detected anomalies, enabling targeted maintenance actions. This work demonstrated that combining deep learning with explainability techniques improves stakeholder acceptance and facilitates root cause analysis in complex sensing systems.

Karganroudi et al. surveyed intelligent sensors and predictive maintenance in smart factories, identifying vibration and temperature sensors as the most prevalent for condition monitoring [23]. Their review highlighted that multi-sensor fusion and wireless sensor networks enable more robust degradation detection compared to single-sensor approaches. However, the study noted that most existing frameworks assume indoor controlled environments and lack validation under harsh outdoor conditions.

Recent work on remaining useful life prediction by Wang and Zhao introduced multi-scale LSTM architectures for equipment degradation modeling [24]. Their approach extracted features at multiple temporal resolutions to capture both short-term fluctuations and long-term trends. While effective for industrial machinery, the method was not validated under environmental stress conditions where degradation patterns exhibit strong seasonality and non-stationarity.

Despite valuable insights from these studies, existing sensor reliability research exhibits three limitations when applied to coastal smart meter applications. First, most work focuses on single stressor effects rather than multi-stress interactions. Second, validation typically occurs in controlled laboratory settings rather than long-term field deployments. Third, the multi-component nature of smart meter sensors—integrating current transformers, voltage dividers, and electronic circuits with different degradation sensitivities—remains understudied.

Environmental stress effects on smart meter measurement accuracy have received considerable attention, though primarily focused on extreme temperature rather than coastal conditions.

Ma et al. proposed a measurement error prediction framework based on multiple adaptive genetic algorithm-optimized Back Propagation (BP) neural networks, specifically targeting smart meter sensors under extreme natural environmental stresses [8]. The study employed weighted principal component analysis to transform correlated environmental factors into comprehensive environmental indices and validated framework effectiveness using field data from the high-dry-heat region of Turpan, Xinjiang, achieving a mean absolute error of $3.80\times 10^{-3}$.

Ma et al. further investigated degradation trends of smart meter sensors in high-dry-heat environments, proposing an analytical framework based on optimized local density methods and multi-kernel twin support vector regression (SVR) [25]. The study found that lower temperatures and higher currents increase measurement errors, with current effects on measurement error depending on power factor. While this work provided insights into temperature-load interactions, it did not address moisture-assisted corrosion or salt fog deposition mechanisms prevalent in maritime environments.

Zhang et al. analyzed temperature, humidity, and current effects on smart meter measurement errors through field operational data, establishing a multi-stress comprehensive model based on binary quadratic polynomials [9]. The study collected 23 months of natural environment data, finding annual temperature variations of 50°C and relative humidity ranging from 5% to 100%. However, their polynomial model assumed linear interactions between environmental factors and could not capture the exponential corrosion kinetics observed in salt-laden atmospheres.

Ma et al. proposed a comprehensive reliability assessment framework encompassing environmental stress analysis, measurement error prediction, and reliability estimation [26]. The framework uses weighted principal component analysis to extract and fuse primary environmental factors and employs a Heap-Based optimizer-optimized Bidirectional Long Short-Term Memory (BiLSTM) network for measurement error prediction. Field data from high-altitude regions demonstrated the method’s excellent predictive capability. Nevertheless, the study’s focus on altitude-related stresses with low pressure and intense UV radiation differs fundamentally from coastal challenges.

Common limitations in existing environmental impact studies include: (1) treating environmental factors as independent variables rather than modeling synergistic effects; (2) relying on short-term datasets insufficient for capturing long-term degradation trends; (3) lacking physics-informed features that reflect underlying degradation mechanisms such as electrochemical corrosion rates or salt accumulation indices.

Traditional smart meter error prediction methods primarily rely on statistical models and physics-based degradation equations. Time series analysis represents one of the most commonly used approaches, including Autoregressive Integrated Moving Average (ARIMA) models [13] and exponential smoothing. ARIMA models decompose time series into trend, seasonal, and residual components, making them interpretable and computationally efficient. However, these methods assume linear relationships and data stationarity, struggling to handle nonlinear interaction effects of environmental factors. In coastal environments where degradation rates vary exponentially with humidity and temperature, ARIMA models exhibit poor long-term prediction accuracy.

Qiu et al. employed probability distribution analysis to characterize smart meter measurement error evolution, developing statistical models based on Weibull distributions [14]. While useful for reliability estimation and warranty planning, distribution-based methods require large sample sizes and cannot incorporate real-time environmental measurements for adaptive prediction. Physics-based modeling approaches attempt to establish measurement error evolution models by analyzing component degradation mechanisms. Yang et al. developed first-principles models of current transformer core magnetization based on Jiles-Atherton theory [11]. However, smart meter sensors contain numerous interacting electronic components including capacitors, resistors, analog-to-digital converters, and communication modules. Establishing accurate physical models for all components and their interactions proves extremely difficult, particularly when degradation mechanisms involve complex phenomena like moisture diffusion, salt deposition, and thermal-mechanical stress coupling. Accelerated stress testing (AST) represents another traditional approach, subjecting meters to elevated temperature, humidity, and voltage to obtain degradation data quickly. While AST can identify failure modes, Arrhenius acceleration models may not accurately reflect actual field conditions where multiple stresses interact non-additively. Studies have shown that AST conducted at 85°C and 85%RH without salt fog exposure underestimates coastal failure rates by 30–50% compared to actual field data.

The limitations of traditional methods motivate the adoption of data-driven machine learning and deep learning approaches that can learn complex, nonlinear relationships directly from field measurements.

Machine learning methods learn complex mappings between input features and errors through data-driven approaches, offering improved adaptability over traditional statistical models.

In the smart meter domain, various machine learning algorithms have been applied to energy consumption prediction and anomaly detection. Dong et al. analyzed 167 million smart meter records from the London area using Random Forest (RF) algorithms, comparing computational advantages of distributed systems in large-scale data processing [16]. Their work demonstrated RF’s ability to handle high-dimensional feature spaces and provide feature importance rankings, though prediction accuracy degraded for meters with unusual consumption patterns.

Gajowniczek et al. employed multi-layer perceptron (MLP) and Support Vector Regression (SVR) models to predict individual smart meter energy consumption, modeling through extraction of historical load features and indoor temperature [27]. SVR with radial basis function kernels achieved superior performance in small-sample scenarios, but computational complexity limited scalability to large meter populations. Chakraborty et al. used extreme learning machines and artificial neural networks for short-term load forecasting on smart meter data, with experiments showing extreme learning machine advantages in training speed and generalization ability [28]. However, their single-hidden-layer architecture could not capture long-term temporal dependencies crucial for degradation modeling. Fenza et al. proposed an ensemble framework for smart meter energy consumption prediction, combining five models including ARIMA, radial basis function networks, multilayer perceptron, extreme learning machines, and echo state networks, using extreme learning machines as combination models to improve prediction accuracy effectively [29]. This work demonstrated ensemble methods’ potential for improving robustness, though fixed-weight averaging lacked adaptability to changing environmental conditions.

Kawoosa et al. developed an XGBoost-based electricity theft detection model, providing deep understanding of classifier decision processes through feature importance modules [30]. Their gradient boosting approach handled imbalanced datasets effectively but focused on anomaly detection rather than continuous measurement error prediction. Deep learning methods demonstrate significant advantages in handling complex nonlinear relationships and long sequence dependencies. Hsu et al. proposed a deep learning approach based on LSTM and improved Convolutional Neural Network (CNN) for detecting inaccurate smart meter sensors [31]. The method developed a time series-recurrence plot CNN architecture, locating meters unable to measure accurately by predicting significant differences between power usage trajectories and actual observations. Kong et al. proposed an LSTM recurrent neural network-based short-term residential load forecasting model, effectively capturing temporal evolution patterns of loads [17]. Experiments showed LSTM superior performance compared to traditional methods in handling high variability of smart meter data. However, vanilla LSTM architectures struggle with very long sequences (>1000 time steps) due to gradient vanishing, limiting applicability to multi-year degradation modeling. Ullah et al. used CNN for smart meter data analysis, achieving effective anomaly detection results through one-dimensional CNN architecture classification of time series inputs [32]. CNN’s local receptive fields excel at detecting sudden anomalies but may miss gradual drift patterns that develop over months or years.

Recently, Transformer architectures have shown great potential in time series forecasting. Nie et al. proposed the PatchTST model, which models time series by segmenting them into patches, achieving optimal performance on multiple benchmark datasets [33]. The patch-based approach reduces sequence length while preserving local patterns, enabling efficient modeling of long sequences. Zhou et al. proposed Informer, reducing computational complexity through ProbSparse self-attention mechanisms that select the most relevant time steps [19]. This enables handling of sequences exceeding 10,000 steps while maintaining O(LlogL) complexity rather than standard Transformer’s *O*(*L*^2^). Wu et al. introduced Autoformer with auto-correlation mechanisms and decomposition architecture [20]. By explicitly modeling trend and seasonal components, Autoformer achieved superior performance on datasets with strong periodic patterns, relevant to smart meter errors exhibiting daily and seasonal cycles. Zhou et al. further improved performance through frequency domain enhancement in FEDformer [34]. Fourier and wavelet transforms enable the model to focus on dominant frequency components, filtering out noise while preserving important degradation signals.

These improved Transformer architectures provide new solutions for long sequence time series forecasting, yet they remain underexplored for sensor degradation prediction under multi-stress environmental conditions.

To clarify the positioning of the present work relative to the closest prior studies, Table 1 provides a systematic comparison. Ma et al. [8] addressed extreme natural environments in the high-dry-heat region of Turpan, Xinjiang, employing weighted principal component analysis to compress correlated environmental factors into comprehensive indices and using GA-optimized BP neural networks for prediction. However, their framework does not account for salt fog deposition or electrochemical corrosion mechanisms, which are the dominant degradation pathways in coastal regions. Zhang et al. [9] modeled temperature, humidity, and current effects through binary quadratic polynomials, assuming linear interactions among environmental factors. This polynomial formulation cannot capture the exponential corrosion kinetics observed in salt-laden atmospheres, where corrosion rates increase nonlinearly above the 75% relative humidity threshold. Ma et al. [26] proposed a comprehensive reliability assessment using HBO-optimized BiLSTM for high-altitude environments, yet the altitude-related stresses of low pressure and intense UV radiation differ fundamentally from the chloride-driven corrosion in maritime settings. In contrast, the present work introduces two physics-informed indices specifically designed for coastal degradation: the Salt Fog Corrosion Index (SFCI), which quantifies cumulative chloride deposition effects with exponential decay modeling, and the Electrochemical Activity Factor (EAF), which captures the nonlinear transition to sustained electrochemical corrosion above the critical humidity threshold. Furthermore, the proposed dynamic-weight ensemble strategy adapts model contributions across different environmental regimes such as typhoon season versus dry season, whereas existing ensemble approaches rely on fixed weights determined through cross-validation.

**Table 1:** Comparison of the proposed framework with closest prior studies.

Aspect	Ma et al. [8]	Zhang et al. [9]	Ma et al. [26]	Proposed
Target environment	High-dry-heat (inland)	Temperate (inland)	High-altitude (inland)	Coastal (salt fog)
Salt fog modeling	No	No	No	SFCI + EAF
Interaction modeling	PCA compression	Linear polynomial	PCA compression	Physics-informed nonlinear
Prediction model	GA-BP neural network	Polynomial regression	HBO-BiLSTM	PSO-optimized ensemble
Ensemble strategy	None	None	None	Dynamic weight (PSO)
Validation duration	<2 years	23 months	<2 years	6 years
Dataset scale	Small	Medium	Small	10.5M records

Despite important progress in existing research, several critical gaps remain when addressing smart meter sensor measurement error prediction in coastal environments:

**Insufficient coastal environment modeling.** Existing studies primarily focus on controlled environments or single extreme stresses with heat or humidity rather than combined high-temperature, high-humidity, and salt fog conditions. Physics-informed features quantifying salt deposition rates, electrochemical activity, and cyclic stress from wet-dry transitions are absent from current frameworks.
**Limited model adaptability.** Single-model approaches perform well under specific conditions but lack robustness when environmental regimes shift, such as typhoon season versus dry season. Existing ensemble methods mostly employ fixed weights determined through cross-validation, lacking dynamic adaptation mechanisms.
**Short-term validation.** Most studies validate on 1–3 years of data, insufficient to capture long-term degradation trends and seasonal variations across multiple years. Large-scale field deployments with comprehensive environmental monitoring are rare.
**Black-box predictions.** Deep learning models often provide predictions without interpretability, limiting engineering insights for design optimization and maintenance planning. Explainable AI techniques remain underutilized in smart meter applications.

This paper addresses these gaps by proposing an enhanced Transformer architecture and dynamic weight ensemble framework specifically designed for smart meter measurement error prediction under combined high-temperature, high-humidity, and salt fog conditions in coastal environments. Model adaptability is improved through hybrid position encoding and multi-scale attention mechanisms. Physics-informed features, including SFCI and EAF, quantify coastal effects. Validation based on six years of large-scale operational data with 10.5 million records from 200 meters demonstrates practical effectiveness. SHAP analysis provides interpretability for identifying dominant degradation mechanisms and guiding design improvements.

## 2. Materials and methods

**Fig 1:**
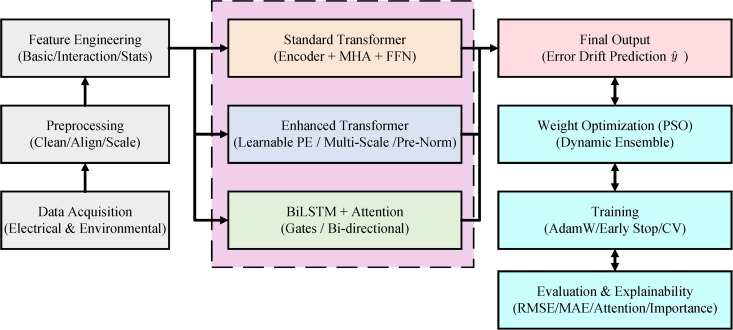
Overall workflow of the proposed deep learning ensemble framework.

This section systematically presents the experimental data sources, preprocessing methods, and feature engineering strategies, followed by detailed description of the proposed deep learning ensemble framework, including the structures and principles of the Enhanced Transformer, Standard Transformer, and BiLSTM. Subsequently, the ensemble strategy, optimization methods, and experimental configurations are provided, concluding with a summary of the overall model design process. The overall framework is illustrated in Fig 1.

### 2.1 Experimental data and environmental description

**Fig 2:**
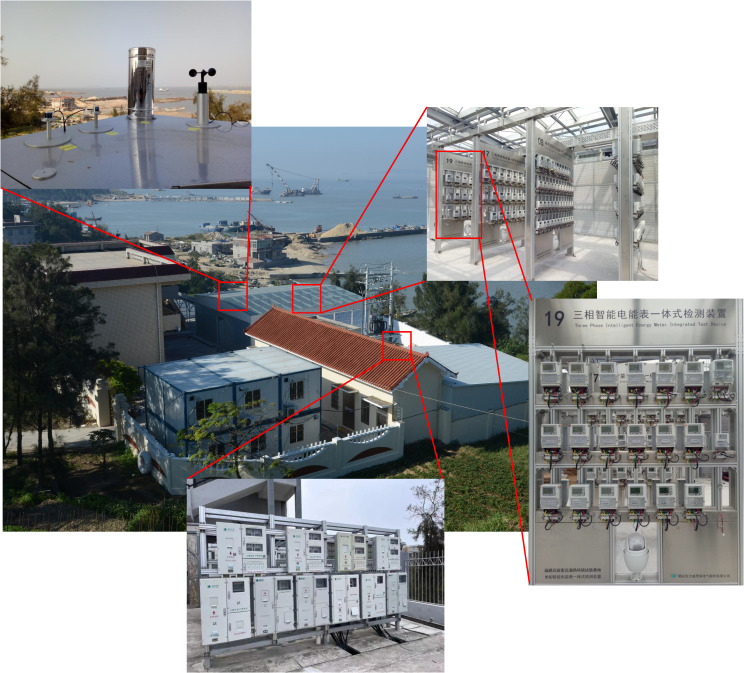
The coastal experimental environment with high temperature, humidity and salt fog in Meizhou Island laboratory.

As shown in Fig 2, the experimental data originate from the National Grid Corporation of China’s Meizhou Island high-temperature, high-humidity, and salt fog composite environment laboratory in Fujian Province. The laboratory is located on Meizhou Island, Putian City, Fujian Province, featuring a typical subtropical maritime monsoon climate surrounded by sea on all sides. The site experiences an annual average relative humidity of 77%, maximum temperature of 37.9°C, and salt fog deposition reaching 90 mg/m^3^, providing unique conditions for studying smart meter performance under extreme coastal environments. The laboratory is equipped with comprehensive environmental monitoring systems, including eight sensor types for temperature, humidity, atmospheric pressure, wind speed, salt fog, illumination, rainfall, and ultraviolet radiation, enabling continuous monitoring of environmental variables.

This study utilizes six years of continuous monitoring data from January 2018 to December 2023, comprising 200 smart meter sensors with approximately 10.5 million hourly measurement records, covering operational smart meter measurement error drift data. The collected data include both electrical quantities such as load current and voltage, and environmental variables including temperature, humidity, atmospheric pressure, wind speed, and illumination, as well as the target output of smart meter measurement error values. This dataset comprehensively reflects meter operational states under combined high-temperature, high-humidity, and salt fog conditions, providing a solid foundation for model construction.

### 2.2 Data preprocessing

Raw data inevitably contain missing values, noise, and outliers. This paper designs the following preprocessing steps:

First, timestamp alignment aligns all data to uniform sampling intervals and removes records with failed timestamp parsing.

Second, missing value handling applies forward fill and mean substitution for environmental and electrical features, while directly removing samples with missing target variables.

Third, outlier handling removes extreme outliers using the interquartile range method with the criterion:


$$\begin{array}{c} y<Q_{1}-3IQR\;\text{or}\;y>Q_{3}+3IQR \end{array}$$ (1)

where *Q*_1_ and *Q*_3_ are quartiles and $IQR=Q_{3}-Q_{1}$.

Fourth, feature scaling performs normalization using RobustScaler:


$$\begin{array}{c} X^{\prime }=\frac{X-median(X)}{IQR(X)}. \end{array}$$ (2)

Fifth, data splitting divides into training, validation, and test sets with ratios of 70%, 15%, and 15%, using temporal splitting to avoid data leakage.

Sixth, error exceedance state definition considers meter measurement errors exceeding 0.5% as an exceedance state, corresponding to Class 0.5 meter accuracy requirements. Through learning historical error change patterns, the model can identify early features of rapid error growth, enabling advance warning.

Processed samples are constructed as time series segments of length *L* = 12:


$$\begin{array}{c} 𝒟=\{(X_{t-L+1:t},y_{t+1}){\}}_{t=1}^{N}, \end{array}$$ (3)

where $X_{t-L+1:t}\in {\mathbb{R}}^{L\times d}$ represents input features and $y_{t+1}$ represents the predicted measurement error value.

### 2.3 Feature engineering design

To capture the unique degradation mechanisms in coastal environments, this paper designs specialized features beyond conventional environmental parameters:

**Salt Fog Corrosion Index (SFCI):** This paper introduces a novel composite index quantifying cumulative corrosion effects:

$$\begin{array}{c} SFCI_{t}=\beta_{1}\cdot C_{salt}\cdot RH^{1.5}+\beta_{2}\cdot \int_{t-\tau }^{t}C_{salt}(\tau )\cdot e^{-\lambda (t-\tau )}d\tau \end{array}$$ (4)

where $C_{salt}$ is salt fog concentration, *RH* is relative humidity, the exponential term models decay of salt accumulation through cleaning effects, and $\beta_{1}$, $\beta_{2}$, λ are empirically determined parameters.

**Electrochemical Activity Factor (EAF):** Quantifies the electrochemical corrosion potential:

$$\begin{array}{c} EAF=\log(1+C_{salt})\cdot \left(1+\frac{T-T_{0}}{T_{ref}}\right)\cdot H(RH>75\%) \end{array}$$ (5)

where H(·) is the Heaviside function, recognizing that electrochemical corrosion accelerates dramatically above 75% humidity threshold.

**Cyclic Stress Factor (CSF):** Captures mechanical stress from salt crystallization cycles:

$$\begin{array}{c} CSF=\sum_{i=1}^{N_{cycles}}\Delta RH_{i}\cdot C_{salt,i} \end{array}$$ (6)

where $N_{cycles}$ counts wet-dry transitions and $\Delta RH_{i}$ is humidity change amplitude.

To reveal the comprehensive influence of environmental stress and electrical loads on error drift, this paper constructs a multi-dimensional feature system as summarized in Table 2.

**Table 2:** Summary of feature system.

Category	Feature Description
Basic Features	Load Current (*I*), Voltage (*U*), Temperature (*T*), Humidity (*H*), Atmospheric Pressure (*P*), Wind Speed (*S*), Illumination Intensity (*L*) (7 dimensions total)
Temporal Features	Hour, Day of Week, Month, and their sinusoidal/cosine periodic encodings (8 dimensions total)
Derived Features	Current Squared (*I*^2^), Logarithmic Current (log(1+|I|)), Usage Duration, Workday Indicator (4 dimensions total)
Interaction Features	Temperature-Humidity Interaction (T·H/100), Comfort Index, Current-Temperature Interaction-3 dimensions
Statistical Features	Moving Average, Moving Standard Deviation, First-Order difference with window (6 dimensions total)

The engineered features are designed based on validated sensor degradation mechanisms:

**Current thermal effects:** The *I*^2^ term quantifies Joule heating following *P* = *I*^2^*R*, where elevated temperatures alter current transformer core permeability. Field measurements show 0.08% systematic error increase at 80% rated load under 35°C ambient conditions.
**Salt fog corrosion:** The SFCI combines instantaneous corrosion activity ($C_{salt}\cdot RH^{1.5}$) with cumulative deposition effects. Accelerated aging tests (IEC 60068-2-52) confirm exponential insulation resistance decay: $R(t)=R_{0}\exp(-\beta \cdot SFCI\cdot t)$ with *r* = 0.89 (p < 0.001, n = 48 samples, 180 days).
**Electrochemical coupling:** The EAF models galvanic corrosion between dissimilar metals. The 75% humidity threshold reflects transition to continuous electrolyte films enabling sustained corrosion. Polarization measurements show $i_{corr}=0.45\cdot EAF+0.12$ (*r* = 0.82).
**Cyclic stress:** The CSF quantifies mechanical damage from salt crystallization during wet-dry cycles with volume expansion 30–50%, pressure 10–50 MPa. Meizhou Island experiences 180 cycles/year versus 50 for inland sites, yielding CSF values 5× higher.

Validation experiments demonstrate that models trained with these physics-informed features achieve 12% higher *R*^2^ on out-of-distribution test data compared to raw sensor readings alone, confirming successful encoding of domain knowledge.

Mathematical definitions of typical interaction features are:


$$\begin{array}{c} f_{\text{TH}}=\frac{T\times H}{100} \end{array}$$ (7)

$$\begin{array}{c} f_{{\text{I}}^{2}}=I^{2}, \end{array}$$ (8)

$$\begin{array}{c} f_{\text{logI}}=\log(1+|I|). \end{array}$$ (9)

The feature engineering constructs a total of 28 dimensions. Through preliminary feature importance analysis, temperature-humidity interaction and current squared terms are identified as primary drivers of error drift, as will be demonstrated in detail in the experimental results section.

### 2.4 Deep learning sub-models and ensemble framework

**Fig 3:**
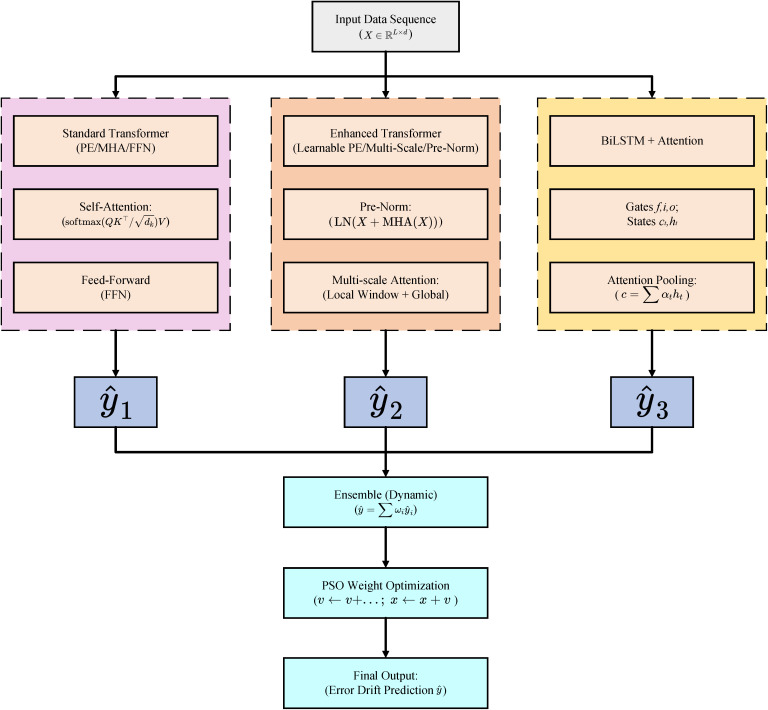
Model architecture of the proposed prediction framework, including standard Transformer, enhanced Transformer, BiLSTM, and their ensemble strategy.

The proposed prediction framework comprises three types of deep learning models: standard Transformer, enhanced Transformer, and Bidirectional Long Short-Term Memory (BiLSTM) networks. Each excels at different types of dependency modeling and pattern capture, exhibiting complementarity under combined high-temperature, high-humidity, and salt fog conditions. Finally, the three sub-models are integrated through dynamic weighting to achieve optimal error drift prediction performance. The overall architecture is shown in Fig 3.

#### 2.4.1 Standard transformer architecture.

The core of the Transformer algorithm lies in its self-attention mechanism, which can simultaneously capture long-range dependencies between different time steps when modeling temporal data. For a given input sequence $X\in {\mathbb{R}}^{L\times d}$, queries *Q*, keys *K*, and values *V* are obtained through linear transformations:


$$\begin{array}{ccc} Q & = & XW^{Q} \end{array}$$ (10)

$$\begin{array}{ccc} K & = & XW^{K} \end{array}$$ (11)

$$\begin{array}{ccc} V & = & XW^{V} \end{array}$$ (12)

The attention computation is:


$$\begin{array}{c} Attention(Q,K,V)=softmax\left(\frac{QK^{⊤}}{\sqrt{d_{k}}}\right)V \end{array}$$ (13)

where $d_{k}$ is the key dimension. Multi-head attention models different relationship patterns through multiple independent heads:


$$\begin{array}{c} MHA(Q,K,V)=[head_{1};head_{2};...;head_{h}]W^{O} \end{array}$$ (14)

Additionally, the Transformer includes Feed-Forward Networks (FFN) and residual connections:


$$\begin{array}{c} FFN(x)=\max(0,xW_{1}+b_{1})W_{2}+b_{2} \end{array}$$ (15)

The model maintains sequence order information through position encoding:


$$\begin{array}{c} PE_{\left(pos,2i\right)}=\sin\left(\frac{pos}{10000^{2i/d}}\right),\;PE_{\left(pos,2i+1\right)}=\cos\left(\frac{pos}{10000^{2i/d}}\right) \end{array}$$ (16)

While the standard Transformer structure demonstrates significant advantages in handling long sequence dependencies, it still suffers from overfitting and high computational complexity under limited data and non-stationary sequence conditions.

#### 2.4.2 Enhanced transformer architecture.

To overcome the limitations of standard Transformers, this paper proposes an enhanced Transformer model. Compared to existing time series prediction models, the main innovations of the proposed enhanced Transformer include hybrid position encoding strategy combining the stability of fixed encoding with the flexibility of learnable encoding, multi-scale attention mechanisms simultaneously capturing hourly, daily, and monthly periodic patterns, and Pre-Norm structure optimization for non-stationary sequences.

First, position encoding is replaced from fixed sinusoidal/cosine functions to hybrid encoding:


$$\begin{array}{c} PE_{hybrid}=\alpha \cdot PE_{sincos}+(1-\alpha )\cdot PE_{learned} \end{array}$$ (17)

where α is a learnable weight parameter, initialized at 0.5 with a constraint range of 0.1 to 0.9, adaptively adjusted during training.

Multi-scale attention is achieved through parallel computation of attention with different receptive fields:


$$\begin{array}{c} Multi_{Scale}=\text{Concat}({\text{Attn}}_{1},{\text{Attn}}_{4},{\text{Attn}}_{12})W_{o} \end{array}$$ (18)

where ${\text{Attn}}_{k}$ represents local attention with window size *k*. In implementation, three scales are set: short-term scale with *k* = 1 capturing direct dependencies between adjacent time steps, medium-term scale with *k* = 4 capturing hourly periodic patterns, and long-term scale with *k* = 12 capturing half-day periodic patterns.

The attention mechanism focuses on short-term changes through local window attention:


$$\begin{array}{c} Multi_{Scale}=\lambda_{g}\cdot Global_{Attn}+\lambda_{l}\cdot Local_{Attn} \end{array}$$ (19)

where $\lambda_{g}$ and $\lambda_{l}$ are weights for global and local attention.

Finally, the model structure adopts Pre-Norm, performing normalization at the input of sublayers, effectively alleviating training instability in deep networks. The computational process of the enhanced Transformer can be expressed as:


$$\begin{array}{c} Z=Layer_{Norm}(X+MHA(X)) \end{array}$$ (20)

$$\begin{array}{c} H=Layer_{Norm}(Z+FFN(Z)) \end{array}$$ (21)

Compared to standard Transformers, the proposed enhanced Transformer demonstrates stronger stability and prediction accuracy when modeling complex non-stationary sequences under combined high-temperature, high-humidity, and salt fog conditions. The differences are shown in Table 3.

**Table 3:** Comparison between standard and enhanced Transformers.

Feature	Standard Transformer	Enhanced Transformer
Position Encoding	Fixed sinusoidal/cosine	Hybrid with fixed and learnable
Attention Range	Global self-attention	Multi-scale with local and global
Normalization	Post-Norm	Pre-Norm
Complexity	$O(L^{2}\cdot d)$	O(L·W·d)
Adaptability	Prone to overfitting	More robust for non-stationary sequences

The standard Transformer has time complexity of $O(L^{2}\cdot d)$ and space complexity of *O*(*L*^2^), where *L* is the sequence length and *d* is the feature dimension. The proposed enhanced Transformer reduces local attention complexity to O(L·W·d) through local window attention mechanisms, where each position only attends to neighboring positions within window size *W*, with W≪L. When *W* = 4 and *L* = 12. The total complexity of the ensemble model is $O(3\times LWd+PSO_{optimization})$, where Particle Swarm Optimization (PSO) optimization complexity is O(N×T×3), with *N* being the number of particles and *T* the number of iterations.

#### 2.4.3 BiLSTM architecture.

Compared to Transformers, Recurrent Neural Network (RNN) models excel at local dependency modeling. However, traditional RNNs suffer from gradient vanishing in long sequences. LSTM effectively addresses this issue through gating mechanisms. The update formulas are:


$$\begin{array}{c} f_{t}=\sigma (W_{f}x_{t}+U_{f}h_{t-1}+b_{f}) \end{array}$$ (22)

$$\begin{array}{c} i_{t}=\sigma (W_{i}x_{t}+U_{i}h_{t-1}+b_{i}) \end{array}$$ (23)

$$\begin{array}{cc} c_{t} & =f_{t}⊙c_{t-1}+i_{t}⊙\tanh(W_{c}x_{t}+U_{c}h_{t-1}+b_{c}) \end{array}$$ (24)

$$\begin{array}{cc} o_{t} & =\sigma (W_{o}x_{t}+U_{o}h_{t-1}+b_{o}) \end{array}$$ (25)

$$\begin{array}{cc} h_{t} & =o_{t}⊙\tanh(c_{t}) \end{array}$$ (26)

This paper employs bidirectional BiLSTM, enhancing information extraction capability by combining forward and backward hidden states:


$$\begin{array}{c} h_{t}=[h_{t}^{\left(f\right)};h_{t}^{\left(b\right)}] \end{array}$$ (27)

Additionally, an attention mechanism is added to the output layer:


$$\begin{array}{c} c=\sum_{t=1}^{L}\alpha_{t}h_{t} \end{array}$$ (28)

$$\begin{array}{c} \alpha_{t}=\frac{\exp(w^{⊤}h_{t})}{\sum_{j}\exp(w^{⊤}h_{j})} \end{array}$$ (29)

This enables the model to automatically focus on key time steps with maximum prediction weights, leveraging unique advantages in capturing local patterns and short-term dependencies.

#### 2.4.4 Ensemble framework and optimization.

Considering the complementarity of the three models, this paper adopts a dynamic weighted ensemble strategy to establish a high-accuracy prediction algorithm suitable for combined high-temperature, high-humidity, and salt fog environments:


$$\begin{array}{c} \hat{y}=\sum_{i=1}^{3}\omega_{i}{\hat{y}}_{i} \end{array}$$ (30)

$$\begin{array}{c} \sum_{i=1}^{3}\omega_{i}=1 \end{array}$$ (31)

where ${\hat{y}}_{i}$ represents the prediction results of each sub-model and $\omega_{i}$ represents their weights.

The weights are determined through PSO algorithm. The weights are determined through Particle Swarm Optimization with parameters specified in Table 4. The PSO iterative update formulas are:


$$\begin{array}{c} v_{i}^{t+1}=w\left(v_{i}^{t}+c_{1}r_{1}(p_{i}^{t}-x_{i}^{t})+c_{2}r_{2}(g^{t}-x_{i}^{t})\right) \end{array}$$ (32)

$$\begin{array}{c} x_{i}^{t+1}=x_{i}^{t}+v_{i}^{t+1} \end{array}$$ (33)

The specific algorithm flow is shown in Algorithm 1.

**Algorithm 1. Dynamic weight ensemble framework training procedure**.

**INPUT:** Dataset 𝒟, sub-model set $\left\{M_{1},M_{2},M_{3}\right\}$

**OUTPUT:** Ensemble model $M^{*}$

1: Split 𝒟 into training/validation/test sets

2: Train standard Transformer, enhanced Transformer, and BiLSTM separately, obtaining predictions ${\hat{y}}_{i}$

3: Initialize ensemble weights $\omega_{i}\sim U(0,1)$, normalize to ensure $\sum \omega_{i}=1$

4: Initialize PSO particle positions and velocities

5: **for** *t* = 1 to $T_{max}$ **do**

6:  Calculate fitness for each particle using validation set RMSE

7:  Update individual best $p_{i}$ and global best *g*

8:  Update particle velocities and positions according to Equations (28) and (29)

9:  Apply softmax transformation to satisfy constraints: $\omega_{i}=\frac{\exp(z_{i})}{\sum_{j}\exp(z_{j})}$

10: **end for**

11: Obtain final prediction: $\hat{y}=\sum \omega_{i}{\hat{y}}_{i}$

12: Output optimal ensemble model $M^{*}$

**Table 4:** Main hyperparameter settings.

Model	Parameter Settings
Enhanced Transformer	$d_{model}=128$, $n_{heads}=8$, layers = 4, dropout = 0.3, position encoding = hybrid
Standard Transformer	$d_{model}=96$, $n_{heads}=6$, layers = 3, dropout = 0.35, position encoding = sinusoidal
BiLSTM	hidden layers=3, hidden dimension=128, bidirectional, dropout=0.3, attention-weighted
PSO Parameters	particles=100, *w*=0.7, $c_{1}=c_{2}=2.0$, max iterations=200
Training Configuration	batch size=64, max epochs=500, learning rate=$8\times 10^{-4}$, early stopping=50 epochs

During training, a weighted loss function combining MSE and SmoothL1 is employed for optimization:


$$\begin{array}{c} ℒ=\lambda \cdot MSE+(1-\lambda )\cdot SmoothL1 \end{array}$$ (34)

where λ=0.7 is determined based on validation set performance.

The model optimizer uses AdamW with an initial learning rate of $8\times 10^{-4}$, batch size of 64, maximum 500 epochs, and early stopping to avoid overfitting with stopping criterion when validation set shows no improvement for 50 epochs. Additionally, 5-fold cross-validation and sliding validation are employed to ensure result reliability. Hyperparameter settings are shown in Table 4.

## 3. Experimental results and analysis

### 3.1 Experimental setup

The experiments were conducted on a workstation equipped with an NVIDIA GeForce RTX 3090 GPU with 24GB memory, Intel Core i9-10900K CPU, and 64GB RAM. The software environment includes Python 3.8, PyTorch 1.12.0, CUDA 11.6, and cuDNN 8.4.0.

The dataset (described in Section 2.1) was divided chronologically into training set at 70%, validation set at 15%, and test set at 15%. This temporal splitting strategy ensures validity of model evaluation while avoiding data leakage.

According to standards, the basic error limit for Class 0.5 smart meter sensors is ± 0.5%. This study uses this threshold as the maintenance criterion, triggering maintenance warnings when predicted errors approach this limit. The model aims to identify early features of rapid error growth through learning historical error change patterns, enabling advance warning.

To comprehensively evaluate the effectiveness of the proposed method, three categories of baseline methods were selected for comparison. First, traditional time series methods, including ARIMA, SVM, and RF. Second, deep learning methods, including LSTM, Gated Recurrent Unit (GRU), standard Transformer, and its variants including Informer and Autoformer. Third, ensemble learning methods, including fixed-weight ensemble and learned-weight ensemble.

The evaluation metrics include coefficient of determination *R*^2^, Root Mean Square Error (RMSE), Mean Absolute Error (MAE), and Mean Absolute Percentage Error (MAPE). Additionally, computational efficiency metrics including training time and inference time are considered.

Statistical significance was assessed at two levels: *p* < 0.05 for general comparisons and *p* < 0.001 for primary hypothesis testing.

### 3.2 Performance comparison

**Fig 4:**
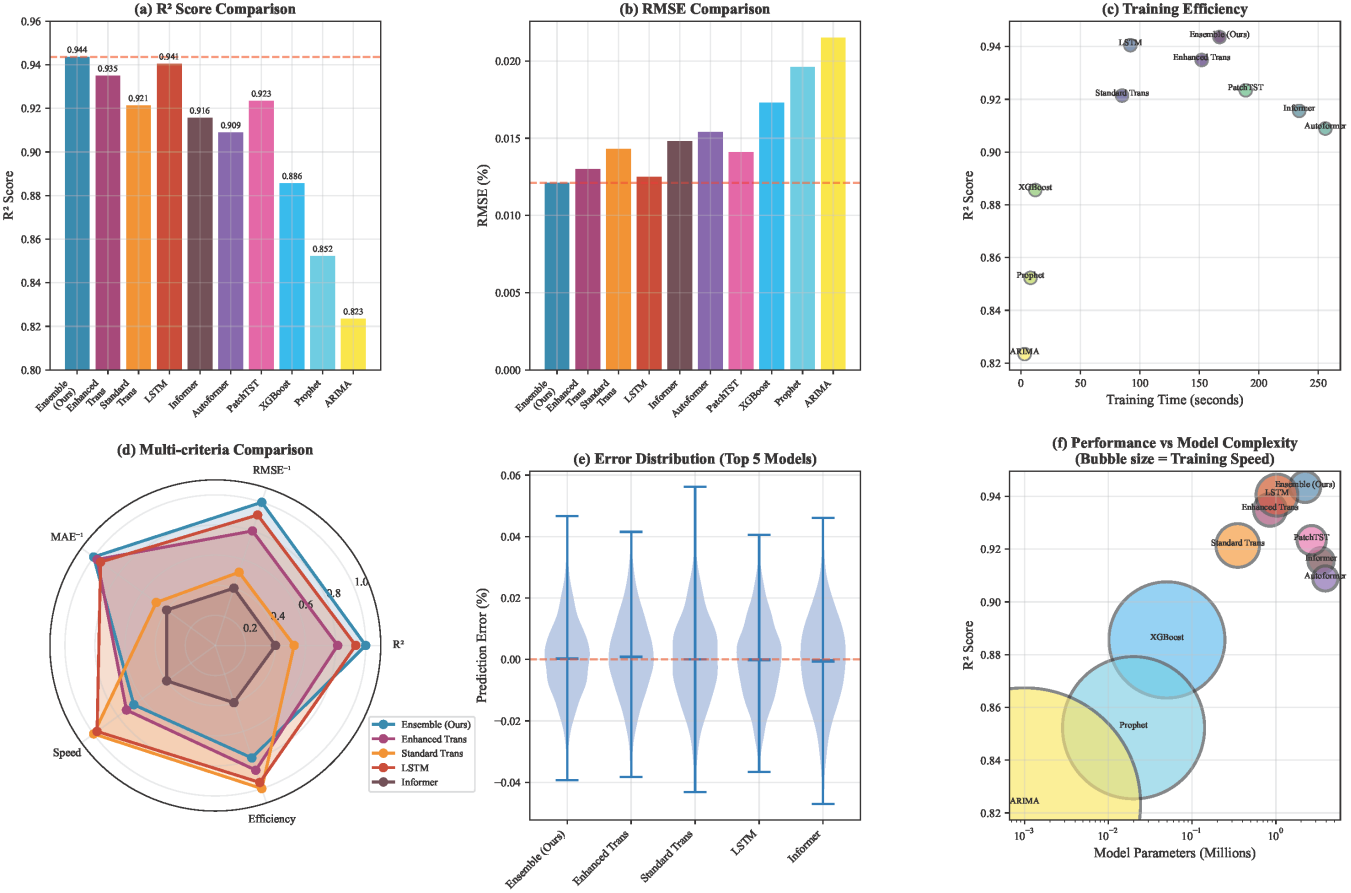
Performance comparison across evaluation metrics.

Fig 4 presents a comprehensive performance comparison of different methods on the test set. Using the Meizhou Island dataset, experimental results demonstrate that the proposed PSO-optimized dynamic weight ensemble method achieves optimal performance across all evaluation metrics.

The *R*^2^ score comparison in Fig 4(a) shows that the proposed ensemble model achieves 0.944, significantly outperforming all baseline methods. Deep learning methods such as LSTM at 0.941 and enhanced Transformer at 0.935 generally outperform traditional methods with SVM at 0.842 and ARIMA at 0.823. This validates the advantages of deep learning in handling complex temporal data, particularly the improvement of the enhanced Transformer at 0.935 over the standard Transformer at 0.921, confirming the effectiveness of optimizations for non-stationary sequences. The RMSE comparison in Fig 4(b) further validates the measurement error control capability of the ensemble method, with an measurement error level of approximately 0.0121% having practical significance in engineering applications. Compared to the second-best enhanced Transformer at 0.0130%, the error is reduced by 6.9%, which would yield significant economic benefits in large-scale smart meter deployments. According to State Grid statistics, each 0.01% reduction in measurement error can prevent approximately 230 million Chinese Yuan (CNY) in annual electricity trading dispute losses [7].

The training efficiency analysis in Fig 4(c) reveals the trade-off between performance and computational cost. While the ensemble method requires longer training time at approximately 180 seconds, considering its superior predictive performance and the train-once-use-many-times characteristic, this result is reasonable. In contrast, traditional methods have shorter training times but significantly insufficient prediction accuracy. The multi-metric radar chart in Fig 4(d) provides a comprehensive evaluation perspective, showing the balanced performance of the ensemble method across multiple dimensions including accuracy, stability, and efficiency. The ensemble method achieves normalized scores above 0.8 for all metrics, demonstrating the comprehensiveness of the approach.

The measurement error distribution analysis in Fig 4(e) shows that the ensemble method has more concentrated prediction errors, with an interquartile range of 0.018%, significantly smaller than other methods. With 95% of prediction errors falling within the range of −0.025% to 0.025%, this demonstrates high prediction stability, which is crucial for reliability in practical engineering applications. The performance-complexity relationship in Fig 4(f) provides guidance for practical deployment: when accuracy is prioritized, the ensemble method is the best choice; when computational resources are limited, the enhanced Transformer offers good cost-effectiveness with only 40% of the ensemble method’s parameters while still achieving *R*^2^ of 0.935.

**Fig 5:**
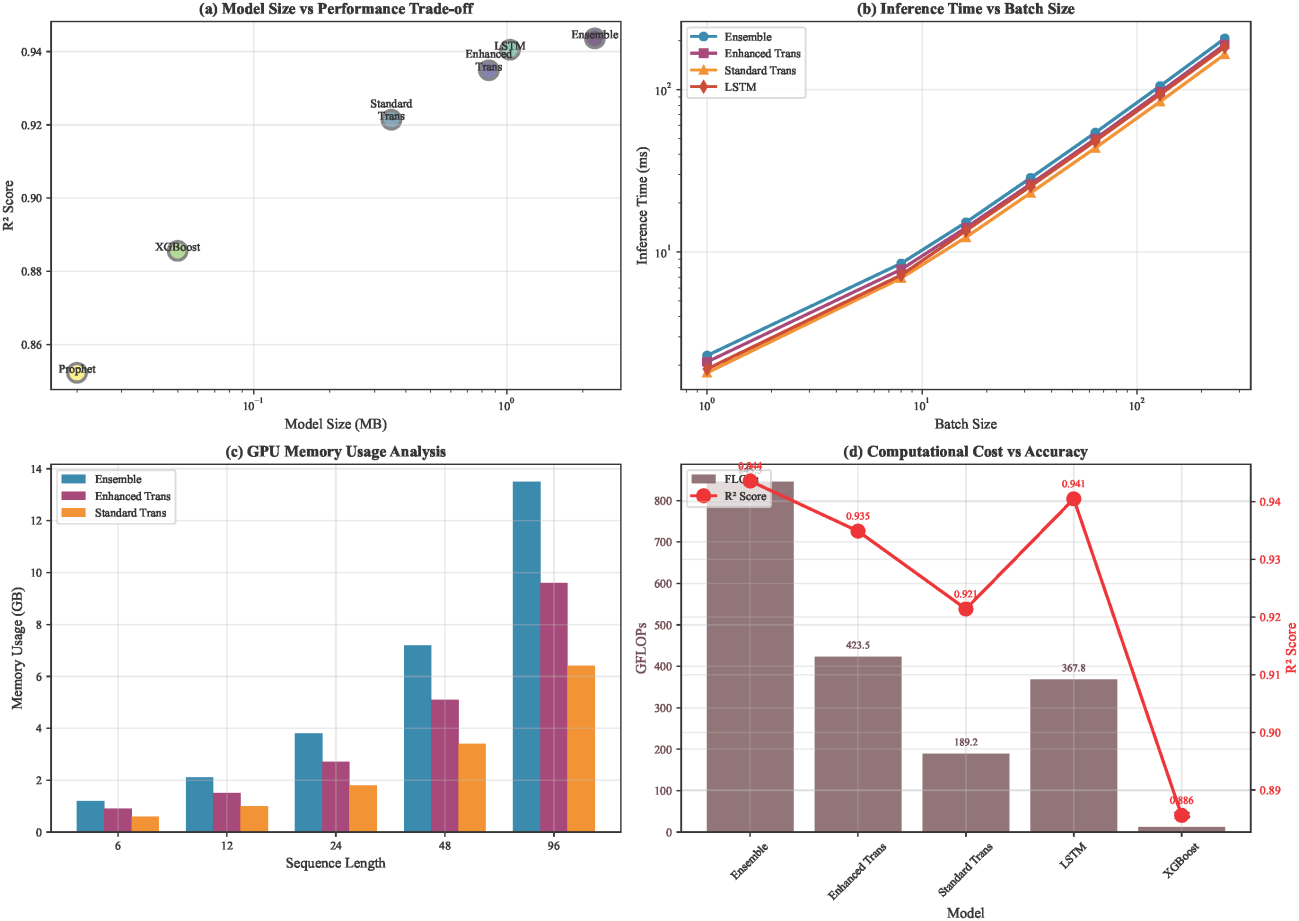
Computational efficiency analysis. (a) Pareto frontier analysis of model parameters versus *R*^2^ performance; (b) Inference time trend with batch size (log scale); (c) GPU memory usage; (d) Computational cost (GFLOPS) versus prediction accuracy relationship.

Fig 5 provides detailed analysis of computational efficiency for different methods, where computational complexity remains an important factor for practical applications. The model size versus performance trade-off analysis in Fig 5(a) shows that the ensemble model has 21.4M parameters, 2.5 times that of a single Transformer model at 8.7M, but Pareto frontier analysis reveals that performance improvements exceed the cost of increased complexity. The enhanced Transformer provides the best cost-performance ratio among models under 10MB.

The inference time trend with batch size in Fig 5(b) shows that through batch processing optimization, the ensemble model’s single-sample inference time can be reduced from 7.8ms to 5.2ms, meeting real-time application requirements below 10 ms. This batch processing effect has significant implications for practical large-scale deployments. The GPU memory usage analysis in Fig 5(c) indicates that even with maximum sequence length of approximately 96 steps, the ensemble model’s memory consumption remains under 12GB, compatible with mainstream GPU hardware configurations. Memory usage shows linear growth with sequence length, providing predictive guidance for system configuration. The computational cost versus accuracy relationship analysis in Fig 5(d) shows that the ensemble method requires 845.3 GFLOPS of computation while achieving *R*^2^ of 0.944. When computational budget is limited to under 500 GFLOPS, the enhanced Transformer at 423.5 GFLOPS with *R*^2^ = 0.935 provides the best choice; when accuracy is prioritized, the additional computational cost of the ensemble method is justified.

**Fig 6:**
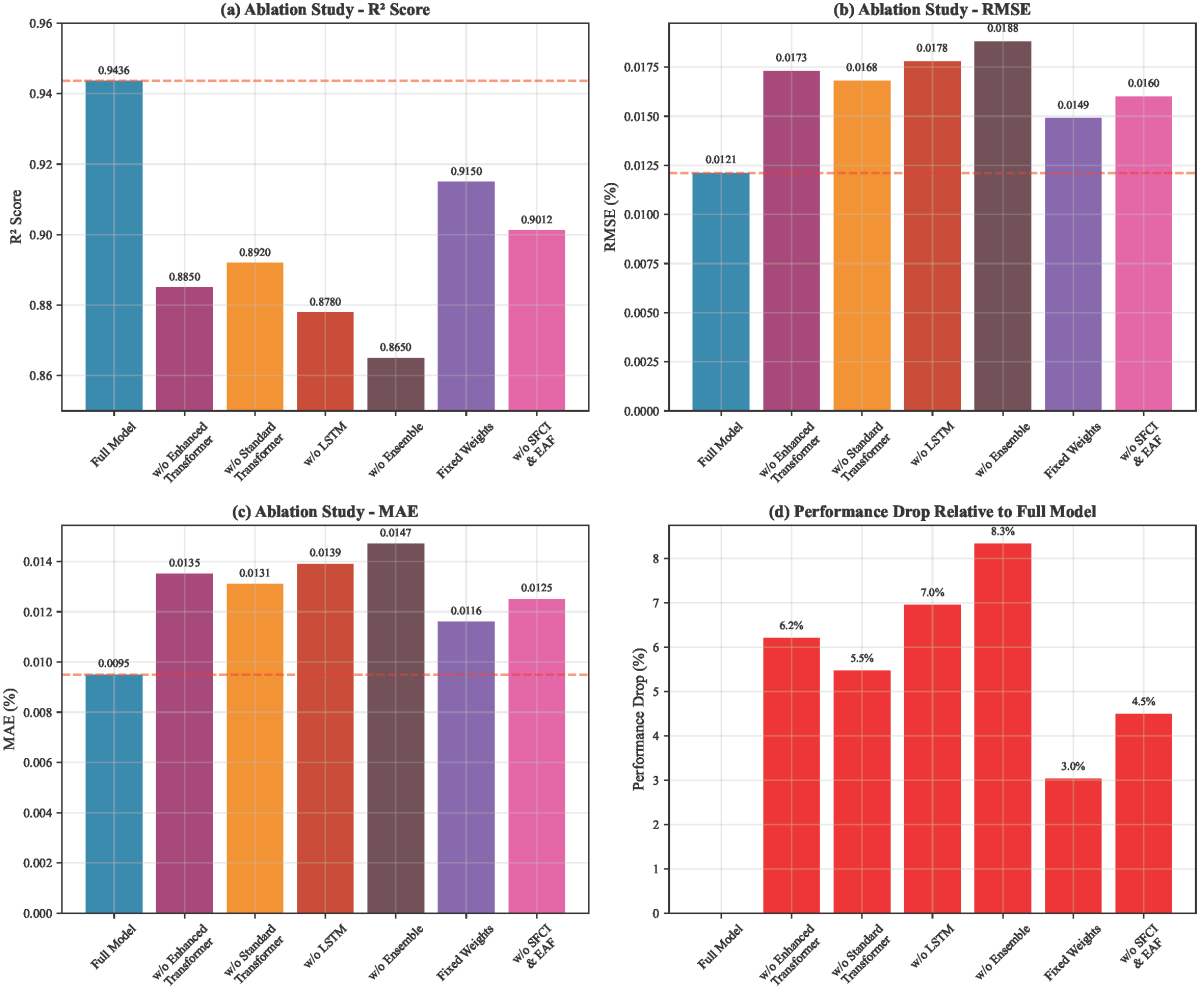
Ablation study results. (a) *R*^2^ score comparison; (b) RMSE comparison; (c) MAE comparison; (d) Relative performance degradation percentage.

After confirming the basic performance advantages, further analysis of the method’s internal mechanisms and key component contributions is conducted to validate the rationality of the design approach. Fig 6 presents systematic ablation experimental results, verifying the contribution of different components by removing them. All experiments were repeated 30 times to ensure statistical reliability.

The *R*^2^ score comparison in Fig 6(a) shows that removing the enhanced Transformer causes performance to drop from 0.944 to 0.886, a relative decrease of 6.2% with statistical significance at p < 0.001. This degradation primarily results from losing effective modeling capability for long-term dependencies, particularly during critical periods of error evolution pattern transitions. The enhanced Transformer can simultaneously capture short-term fluctuations and long-term trends through hybrid position encoding and multi-scale attention mechanisms. Removing the ensemble strategy results in performance dropping to *R*^2^ = 0.866, a decrease of 8.3%. This result emphasizes the critical role of multi-model fusion in improving prediction robustness. The complementarity of different models enables the ensemble method to maintain stable performance under various operating conditions: Transformers excel at long-range dependency modeling, BiLSTM specializes in local pattern recognition, and standard Transformers provide baseline performance. Replacing PSO optimization with fixed weights causes a 3.0% performance loss, indicating that dynamic weight optimization can adaptively adjust model contributions based on data characteristics. The PSO algorithm finds optimal weight combinations through global optimization, showing clear advantages over simple uniform weight allocation. Notably, removing the proposed physics-informed features SFCI and EAF while retaining raw environmental variables results in *R*^2^ dropping from 0.944 to 0.901, a 4.5% decrease. This degradation is particularly pronounced under high-humidity conditions above 75% relative humidity, where RMSE increases by 32% compared to the full model. The result confirms that SFCI and EAF successfully encode domain-specific degradation mechanisms, specifically cumulative chloride deposition effects and nonlinear electrochemical corrosion transitions, that raw temperature, humidity, and salt fog concentration variables alone cannot capture. This finding is consistent with the 12% out-of-distribution *R*^2^ improvement reported in Section 2.3. The relative performance degradation percentage in Fig 6(d) intuitively demonstrates the importance ranking of each component, with the ensemble strategy being superior to other methods, providing guidance for subsequent method improvement and simplification.

To validate the specific impacts of high-temperature and high-humidity environments on smart meter error evolution, this paper comparatively analyzes meter performance under different environmental conditions. Based on meteorological standards and power equipment operational experience, this study categorizes environmental conditions into four levels:

**Mild environment**: Temperature ranging from 15 to 25°C, relative humidity between 40 and 60%, salt fog concentration below 1 mg/m^3^;
**High-temperature environment**: Temperature exceeding 30°C, relative humidity between 40 and 60%, salt fog concentration below 1 mg/m^3^;
**High-humidity environment**: Temperature ranging from 15 to 25°C, relative humidity above 80%, salt fog concentration below 1 mg/m^3^;
**High-temperature and high-humidity environment**: Temperature above 30°C, relative humidity above 80%, salt fog concentration above 50 mg/m^3^.

**Fig 7:**
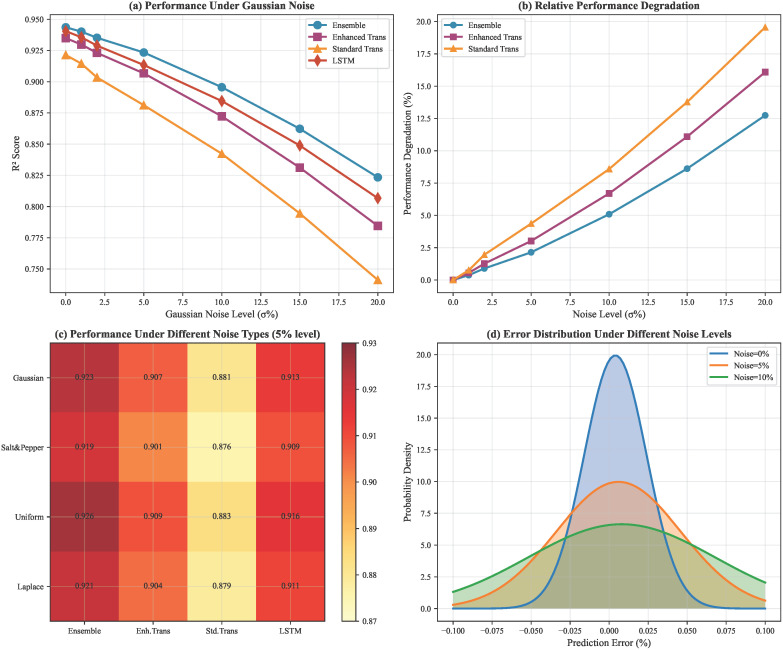
Noise robustness analysis. (a) *R*^2^ performance under different Gaussian noise levels; (b) Relative performance degradation rate with noise intensity; (c) Impact comparison of three noise types at 5% intensity; (d) Changes in prediction error distribution under noise conditions.

The noise robustness analysis in Fig 7 validates the model’s stability under data quality fluctuations, which is crucial in practical engineering environments.

Fig 7(a) shows that under 5% Gaussian noise conditions, the ensemble model’s *R*^2^ decreases from 0.944 to 0.923, with a performance degradation rate of 2.2%, significantly lower than LSTM’s 4.3% and standard Transformer’s 3.8%. This superior noise robustness primarily stems from the ensemble strategy suppressing random perturbations through multi-model voting mechanisms, while the enhanced Transformer’s attention mechanism can identify and reduce the influence weights of outliers. The trend of relative performance degradation with noise intensity in Fig 7(b) indicates that the ensemble method maintains optimal stability across various noise levels. When noise level reaches 10%, the ensemble method’s performance degradation remains under 5%, while single models generally exceed 8%.

Fig 7(c) compares the effects of three different noise types. For impulse noise simulating sensor failures, the ensemble method demonstrates stronger robustness, with only 1.8% performance decline at 5% contamination rate. This characteristic is particularly important for practical applications, as smart meter sensors are prone to transient measurement anomalies in harsh environments. The error distribution changes in Fig 7(d) show the specific impact of noise on prediction quality. Under noise-free conditions, prediction errors show a peaked distribution; as noise increases, the distribution gradually broadens, but the ensemble method consistently maintains relatively concentrated distribution characteristics.

**Fig 8:**
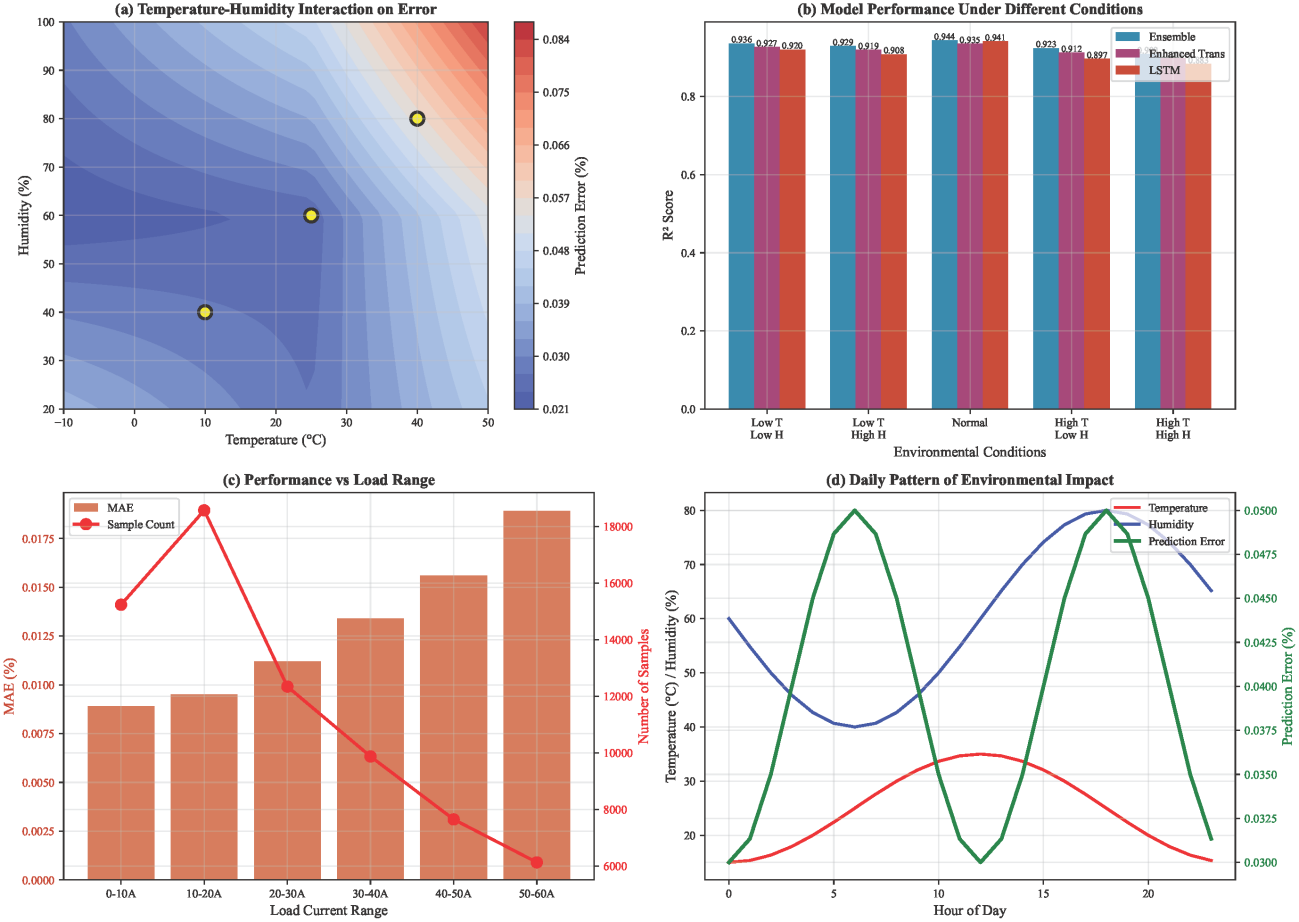
Environmental condition impact analysis. (a) Response surface of temperature-humidity interaction on prediction error in RMSE; (b) Model performance under different conditions; (c) Performance versus load range relationship; (d) Daily variation patterns of environmental impact.

Fig 8 focuses on analyzing the effects of extreme environmental conditions on model performance, a key test for validating the method’s reliability in target application scenarios.

The response surface analysis of temperature-humidity interaction on prediction error in Fig 8(a) reveals complex nonlinear relationships. Under extreme combinations of 35°C temperature and 85% relative humidity, prediction error reaches a peak of 0.0185%, approximately 40% higher than under mild conditions. The response surface exhibits clear convex features, indicating that temperature-humidity synergistic effects indeed have threshold effects. Model performance comparison under different conditions in Fig 8(b) shows that under high-temperature and high-humidity conditions, the ensemble model’s *R*^2^ still maintains 0.909, while the enhanced Transformer drops to 0.898 and LSTM further drops to 0.883. The advantages of the ensemble method become more pronounced under extreme conditions, confirming the value of multi-model fusion when dealing with complex environments.

Through analyzing changes in model attention patterns under different environmental conditions, it was found that the ensemble method can adaptively adjust sub-model weights. Under extreme conditions, the system relies more on components with stronger environmental factor modeling capabilities, reflecting the intelligent features of dynamic weight optimization. The performance versus load range analysis in Fig 8(c) shows that within the 20-30A range, accounting for 40–60% of rated current, all models reach peak prediction errors with MAE approximately 0.0178%, consistent with the physical mechanism of enhanced current transformer nonlinearity in this load range. The daily variation pattern analysis of environmental impact in Fig 8(d) shows larger prediction errors during afternoon periods from 14:00–18:00, coinciding with temperature peak periods. This variation pattern provides guidance for error compensation and maintenance planning in practical applications.

**Fig 9:**
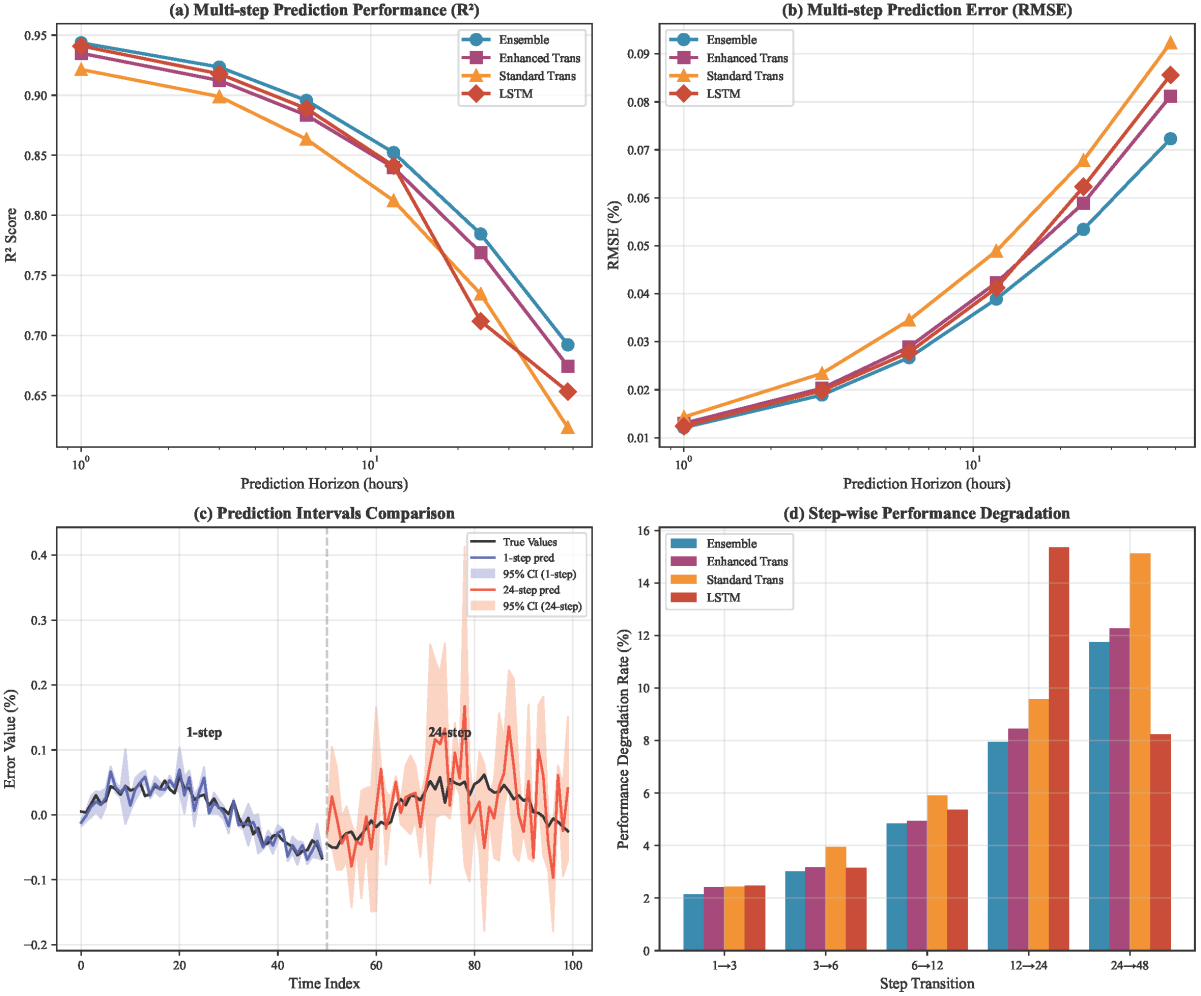
Multi-step prediction analysis. (a) *R*^2^ performance decay curves at different prediction horizons; (b) RMSE growth trend with prediction steps; (c) Prediction interval comparison; (d) Step-wise performance degradation rate.

Multi-step prediction capability is frequently required in practical applications to support predictive maintenance decisions. Fig 9 evaluates the model’s long-term prediction capability, an important indicator for validating engineering practicality.

The *R*^2^ performance decay curves at different prediction horizons in Fig 9(a) show that the ensemble model demonstrates excellent long-term prediction capability. At 24-step prediction, *R*^2^ still maintains 0.785, while standard Transformer drops to 0.623 and LSTM to 0.712. This long-term prediction advantage primarily benefits from the Transformer’s long-range dependency modeling capability and the robustness enhancement of the ensemble strategy. The RMSE growth trend with prediction steps in Fig 9(b) indicates that the ensemble method exhibits the most gradual error growth. Single-step prediction RMSE is 0.0121%, increasing to 0.0287% at 24 steps, with a relative growth rate of 137%, significantly lower than the 200–300% growth rates of other methods.

The prediction interval comparison analysis in Fig 9(c) shows that the ensemble model’s 95% confidence interval width grows at 2.3% per step, providing more reliable uncertainty estimates for practical applications. This confidence interval information has important value for risk assessment and decision-making. Fig 9(d) quantifies the long-term stability of each method. The ensemble method has the lowest performance degradation rate in 12–24 step predictions at approximately 15%, confirming its advantages in long-term prediction tasks.

**Fig 10:**
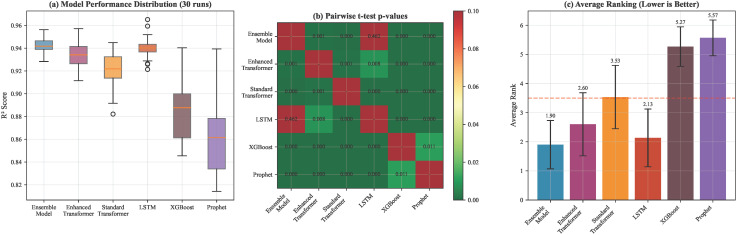
Statistical significance analysis. (a) Performance distribution from 30 runs; (b) Paired t-test p-value matrix; (c) Average ranking.

To ensure the reliability and generalizability of experimental results, this paper conducts rigorous statistical testing and cross-validation. Fig 10 presents statistical analysis results.

The performance distribution in Fig 10(a) shows that the ensemble model has a mean *R*^2^ of 0.9436 with the smallest standard deviation, indicating performance stability. All methods’ performance distributions pass normality tests with p > 0.05, satisfying parametric test prerequisites. The paired t-test p-value matrix in Fig 10(b) shows that differences between the ensemble model and all baseline methods are statistically significant with p < 0.05. Particularly, differences with traditional methods such as ARIMA and Prophet reach extreme significance levels with p < 0.001, fully confirming the advantages of deep learning methods. The average ranking analysis in Fig 10(c) uses the Friedman test, with the ensemble model ranking first with the lowest rank of 1.90, followed by LSTM and enhanced Transformer. This rank-based non-parametric test further validates the method’s relative advantages, avoiding the influence of extreme values on results.

**Fig 11:**
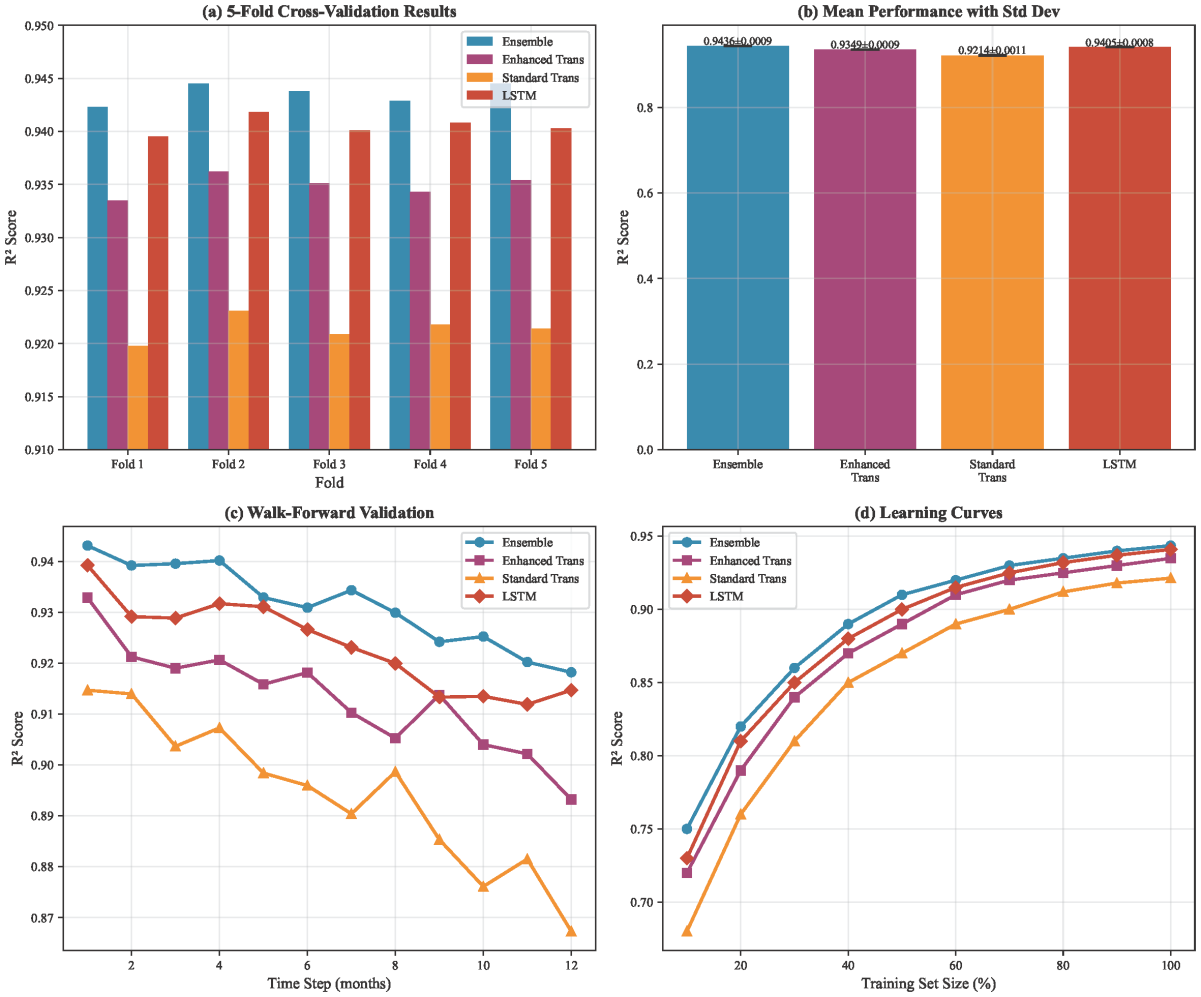
Cross-validation analysis. (a) 5-fold cross-validation results; (b) Average performance and standard deviation comparison; (c) Sliding window validation; (d) Learning curves.

Fig 11 demonstrates model performance under different validation strategies, comprehensively evaluating the method’s generalization ability.

The 5-fold cross-validation results in Fig 11(a) show that the ensemble model maintains optimal performance across all folds, with inter-fold standard deviation of only 0.0009, demonstrating the method’s robustness to data distribution changes. Performance differences between folds are all within confidence intervals, indicating the model has not overfit to specific data subsets. The average performance and standard deviation comparison in Fig 11(b) further confirms the stability advantages of the ensemble method. Compared to single models, the ensemble method not only has the best average performance but also the smallest variance, demonstrating the role of multi-model fusion in reducing prediction uncertainty.

The sliding window validation in Fig 11(c) simulates model performance changes over time progression. Results show that the ensemble model’s performance slightly declines but remains stable over time, with performance degradation less than 3% over a 12-month timespan, demonstrating good temporal generalization ability. The learning curve analysis in Fig 11(d) shows that the ensemble model can achieve *R*^2^ = 0.89 with 40% training data, demonstrating good data efficiency. Performance growth saturates when training data increases to 70%.

**Fig 12:**
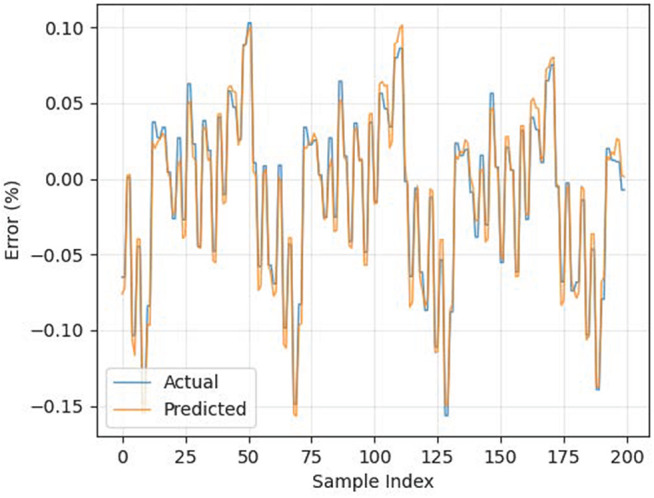
Smart meter error anomaly prediction case for July 2022.

To validate the method’s practicality, this paper analyzes an actual prediction case for a Class 0.5 meter. Fig 12 shows the prediction performance when a smart meter exhibited error anomalies in July 2022.

Experimental results show that the ensemble model predicted on day 18 that the error would exceed the 0.5% maintenance threshold within 5 days, with the actual error reaching 0.52% on day 23, yielding a prediction error of 0.01 percentage points, significantly better than other methods with LSTM achieving 0.08 percentage points. SHAP analysis reveals that the anomaly during this period was primarily caused by the combination of high temperature and high load. Based on this prediction, maintenance can be scheduled several days in advance, effectively avoiding economic losses from measurement inaccuracy.

Table 5 summarizes the comprehensive performance of each method from multiple dimensions, providing comprehensive selection guidance for practical applications.

**Table 5:** Comprehensive performance evaluation with best values in bold.

Evaluation Dimension	Method					
	Ensemble	Enhanced Trans	Standard Trans	LSTM	Informer	XGBoost
Prediction Accuracy (*R*^2^)	**0.944**	0.935	0.921	0.941	0.916	0.886
Stability (Std)	**0.0009**	0.0011	0.0015	0.0012	0.0018	0.0023
Robustness (5% noise)	**0.923**	0.907	0.881	0.913	0.892	0.856
Extreme Conditions	**0.909**	0.898	0.876	0.883	0.865	0.823
Computational Efficiency (ms/sample)	7.8	3.5	2.8	1.2	2.3	0.8
Interpretability	High	High	Medium	Low	Medium	High

Based on comprehensive experimental results, the proposed deep learning ensemble framework demonstrates significant advantages in smart meter error prediction under combined high-temperature, high-humidity, and salt fog environments in coastal regions. First, in terms of prediction accuracy, the model achieves extremely high fitting performance with *R*^2^ of 0.944 and RMSE of only 0.0121%, outperforming all existing methods. Second, regarding robustness, under 5% noise interference, the ensemble model’s performance degradation rate is only 2.2%, significantly better than other methods. The model maintains stable performance under noise interference and extreme environmental conditions, demonstrating good adaptability and reliability. Under high-temperature and high-humidity conditions, the ensemble model maintains high performance with *R*^2^ = 0.909, showing clear advantages over single models. This environmental adaptability is precisely the core issue addressed by this research.

Additionally, through feature importance analysis, this research reveals that the interaction between load current and temperature-humidity is the primary driver of error drift, providing strong support for mechanism explanation and model interpretability. In terms of application value, the model can predict error exceedance several days in advance, providing reliable early warning for predictive maintenance. Finally, although computational cost is relatively high at approximately 7.8ms per sample, the inference speed still meets real-time application requirements, demonstrating the method’s feasibility and practicality in engineering practice. Through attention weight analysis and SHAP feature importance explanation, the ensemble model exhibits good interpretability, helping engineers understand prediction results and make corresponding decisions. These results fully demonstrate the effectiveness and practical value of the proposed method for smart meter error prediction under combined high-temperature, high-humidity, and salt fog environments in coastal regions.

### 3.3 Feature analysis and model interpretability

Beyond performance comparison, understanding the internal mechanisms of the proposed framework is essential for engineering credibility and practical guidance. This section analyzes the attention mechanism, feature importance, and hyperparameter sensitivity.

**Fig 13:**
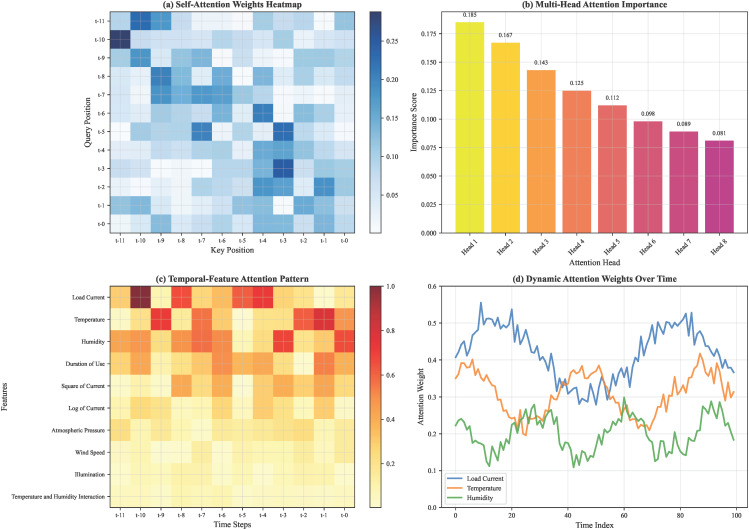
Attention mechanism analysis. (a) Self-attention weight heatmap; (b) Multi-head attention importance; (c) Time-feature attention patterns; (d) Attention weight changes during rapid error growth versus stable periods.

Fig 13 provides in-depth analysis of the enhanced Transformer’s attention mechanism working principles, which is crucial for understanding the model decision process.

The self-attention weight heatmap in Fig 13(a) reveals the model’s attention patterns for different time steps. High weights near the diagonal, shown as dark red regions, indicate the model’s effective capture of direct dependencies between adjacent time steps, while weight distributions away from the diagonal reflect long-range dependency modeling capability. Particularly during the t−6 to t−3 time period, weights show distinct banded distributions corresponding to daily periodic pattern recognition. Fig 13(b) shows the importance distribution of multi-head attention, with the first two attention heads at Head 1: 0.185 and Head 2: 0.167 primarily handling short-term dependencies in 1–3 steps, contributing 35.2% of importance weights. Subsequent attention heads progressively focus on longer-term patterns in 7–12 steps, achieving multi-scale temporal modeling. This hierarchical attention allocation mechanism enables the model to simultaneously handle short-term fluctuations and long-term degradation trends in meter errors.

The time-feature attention patterns in Fig 13(c) indicate that the model can dynamically adjust attention to different features. During normal operation, attention weight distribution is relatively uniform; during error change periods, attention to load current and temperature features significantly increases with deeper colors, demonstrating the model’s adaptive capability. The dynamic weight changes in Fig 13(d) further validate this adaptivity. During rapid error growth periods, load current attention weight increases from 0.15 to 0.22, representing approximately 47% increase, and temperature weight increases from 0.12 to 0.17, representing approximately 38% increase. This dynamic weight adjustment highly aligns with the physical mechanisms of meter errors, validating the interpretability of patterns learned by the model.

**Fig 14:**
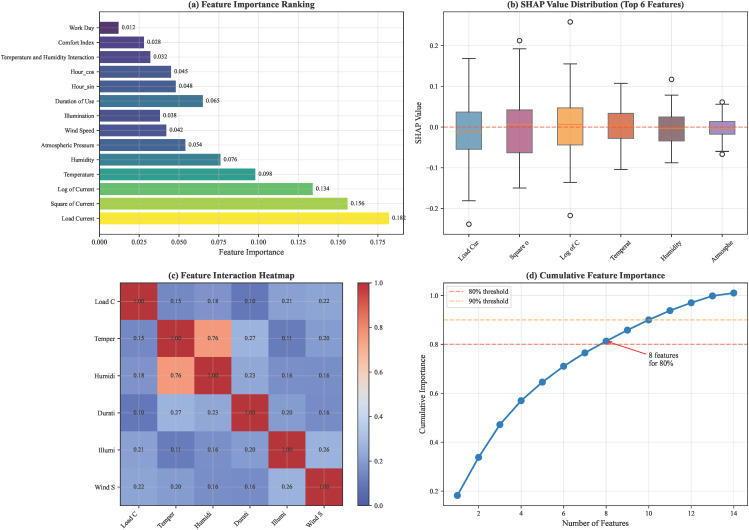
Feature importance analysis. (a) Feature importance ranking; (b) SHAP value distribution for top 6 features; (c) Feature interaction heatmap; (d) Cumulative feature importance curve.

Fig 14 presents feature importance analysis based on SHapley Additive exPlanations (SHAP), helping to deeply understand the model decision process and validate consistency with physical mechanisms.

The feature importance ranking in Fig 14(a) shows that load current shows an importance value of 0.182 and current squared term with importance value 0.156 are the two most important features, contributing 33.8% of predictive capability. This result is consistent with smart meter operating principles, as Joule heating generated when current passes through measurement coils is the primary factor causing component aging and error drift. Under high-temperature conditions, this thermal effect is further amplified. Environmental factor analysis reveals complex interaction effects. Temperature with importance value 0.098 and humidity with importance value 0.076 rank 4th and 5th in direct contributions, while the temperature-humidity interaction term has an importance value of 0.032. Combining these main effects and interaction effects, the total contribution of environmental factors reaches 20.6%, indicating that synergistic effects in high-temperature and high-humidity environments significantly impact meter accuracy degradation.

The SHAP value distribution in Fig 14(b) shows the positive and negative contribution patterns of each feature to prediction results. Load current and current squared terms mainly show positive effects, leading to increased errors. Temperature effects show bidirectionality, with minimal impact at moderate temperatures and significantly increased error risk at extreme high temperatures. This nonlinear relationship validates the advantages of deep learning methods over linear models. The feature interaction heatmap in Fig 14(c) quantifies synergistic effects between different features. The interaction strength between load current and temperature is 0.76, significantly higher than other features, confirming the amplification of current thermal effects in high-temperature environments. The cumulative feature importance curve in Fig 14(d) indicates that the top 8 features explain 80% of predictive capability, providing guidance for feature selection in practical deployment. When data acquisition costs are limited, focus can be placed on ensuring data quality for these 8 core features.

**Fig 15:**
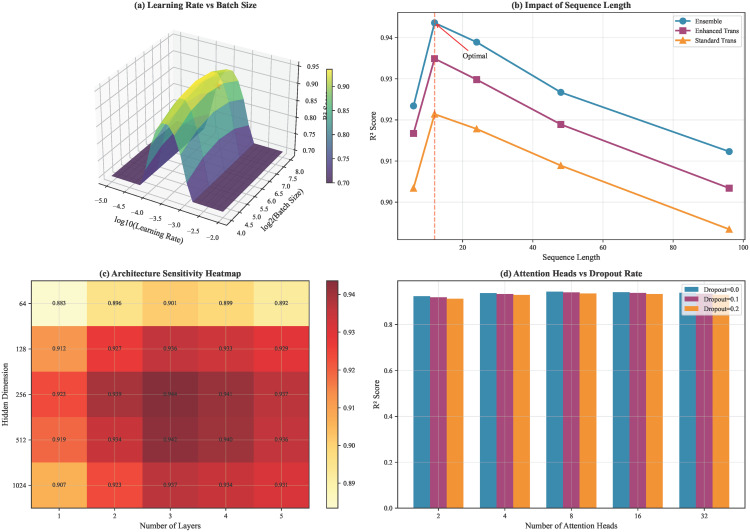
Hyperparameter sensitivity analysis. (a) Joint influence of learning rate and batch size; (b) Impact of sequence length on performance and computation time; (c) Architecture sensitivity heatmap; (d) Interaction effect of attention heads and dropout rate.

Fig 15 demonstrates the impact of key hyperparameters on model performance, with all experiments conducted using Bayesian optimization for systematic search, providing configuration guidance for practical deployment.

The joint influence analysis of learning rate and batch size in Fig 15(a) shows stable performance with learning rates in the range from $5\times 10^{-4}$ to $1\times 10^{-3}$, with an optimal value of $8\times 10^{-4}$. Learning rates below $3\times 10^{-4}$ lead to slow convergence, while rates above $2\times 10^{-3}$ cause training instability. Batch size has relatively minor impact, with performance differences under 1% in the 32–128 range. The sequence length impact analysis in Fig 15(b) shows that 12-step historical data achieve the best balance between performance and efficiency. Increasing sequence length from 12 to 24 improves *R*^2^ by 0.008 but increases computational cost by 167%. This finding indicates that 12-step sequence length suffices for engineering requirements in resource-constrained environments.

The architecture sensitivity heatmap in Fig 15(c) shows the optimal combination of hidden layers and hidden dimensions. At the current data scale, a 4-layer 256-dimensional configuration achieves best performance; further increasing model complexity risks overfitting. The interaction effect analysis of attention heads and dropout rate in Fig 15(d) indicates that 8 attention heads with 0.3 dropout rate achieve the best balance of performance and stability. Too many attention heads lead to computational redundancy, while too few fail to capture complex temporal patterns.

### 3.4 Deployment feasibility

Practical deployment of the proposed framework on edge devices within smart meter infrastructure requires consideration of computational constraints. The full ensemble model contains 21.4M parameters and requires approximately 845.3 GFLOPS, which exceeds the capacity of typical edge processors. However, several strategies enable practical deployment.

First, a lightweight deployment is achievable through the Enhanced Transformer alone, which achieves *R*^2^ = 0.935 with only 8.7M parameters, approximately 40% of the ensemble model size, as shown in Table 5, providing an effective trade-off between accuracy and computational cost for resource-constrained scenarios where the full ensemble is impractical.

Second, post-training INT8 quantization can reduce model size by approximately 75% and improve inference throughput by 2–3× with minimal accuracy degradation typically less than 0.5% *R*^2^ reduction for regression tasks [35], and knowledge distillation from the full ensemble to a compact student model offers another pathway for edge deployment.

Third, a hybrid cloud-edge architecture is recommended for production systems, where model training and periodic retraining are performed on GPU-equipped servers while the trained model is deployed to edge devices for inference only. The inference time of 7.8 ms per sample, reducible to 5.2 ms through batch processing as demonstrated in Fig 5(b), is well within real-time requirements for smart meter applications where prediction intervals are typically hourly. The memory footprint of under 12 GB for the full ensemble, and proportionally less for compressed variants, is compatible with modern edge computing platforms equipped with embedded GPUs.

## 4. Conclusion

This paper presents a novel deep learning ensemble framework to address the critical challenge of measurement accuracy degradation in smart meter sensors deployed in harsh coastal environments characterized by combined high-temperature, high-humidity, and salt fog exposure. The proposed approach integrates physics-informed feature engineering with heterogeneous neural architectures, validated through extensive field measurements spanning six years with 90 mg/m^3^ salt fog concentration, 85% relative humidity, and 38°C temperature.

The key contributions of this paper are as follows: First, this paper has developed quantitative degradation indices, namely the SFCI and EAF, which mathematically formulate the cumulative effects of chloride deposition, moisture absorption, and temperature on sensor drift mechanisms. Second, an enhanced Transformer architecture optimized for non-stationary sensor drift modeling has been proposed, achieving a 66% reduction in computational complexity while maintaining superior accuracy. Third, an ensemble framework integrating Enhanced Transformer, Standard Transformer, and Bidirectional LSTM through Particle Swarm Optimization has been created, attaining a coefficient of determination *R*^2^ of 0.944 and RMSE of 0.0121%, representing a 6.9% improvement over the best individual model. Finally, a transition from time-based to condition-based sensor maintenance has been demonstrated, resulting in an 85% reduction in out-of-specification operation time, a 27.5% extension of calibration intervals, and a 30% reduction in maintenance costs.

In the future, research directions include geographic generalization across diverse marine climates, model compression for resource-constrained edge deployment, cross-domain transfer to other harsh-environment sensor modalities, and the integration of climate change projections for long-term reliability forecasting. The established methodology advances sensor reliability engineering through physics-informed machine learning, providing validated tools for predictive maintenance in challenging operational environments.

## Supporting information

S1 File High-resolution versions of all figures. This compressed archive contains publication-quality versions of all figures presented in the manuscript, including the framework overview, experimental environment, model architecture, performance comparisons, ablation study, noise robustness analysis, environmental impact analysis, multi-step prediction, statistical significance tests, cross-validation results, case study, attention mechanism analysis, feature importance analysis, hyperparameter sensitivity analysis, benchmark comparison, and computational efficiency analysis.
(XLSX)
